# Empowering Implementation Teams with a Learning Health System Approach: Leveraging Data to Improve Quality of Care for Transient Ischemic Attack

**DOI:** 10.1007/s11606-020-06160-y

**Published:** 2020-09-01

**Authors:** Nicholas A. Rattray, Teresa M. Damush, Edward J. Miech, Barbara Homoya, Laura J. Myers, Lauren S. Penney, Jared Ferguson, Brenna Giacherio, Meetesh Kumar, Dawn M. Bravata

**Affiliations:** 1 Department of Veterans Affairs (VA) Health Services Research and Development (HSR&D) Precision Monitoring to Transform Care (PRISM) Quality Enhancement Research Initiative (QUERI), Indianapolis, IN USA; 2 VA HSR&D Center for Health Information and Communication (CHIC), Richard L. Roudebush VA Medical Center, Indianapolis, IN USA; 3 Department of Anthropology, Indiana University-Purdue University, Indianapolis, IN USA; 4 Regenstrief Institute, Inc., Indianapolis, IN USA; 5 Department of Internal Medicine, Indiana University School of Medicine, Indianapolis, IN USA; 6 Veterans Evidence-Based Research Dissemination and Implementation Center (VERDICT), South Texas Veterans Health Care System, San Antonio, TX USA; 7 University of Texas Health San Antonio, San Antonio, TX USA; 8 Office of Healthcare Transformation (OHT), Veterans Health Administration (VHA), Washington, DC USA; 9 Department of Neurology, Indiana University School of Medicine, Indianapolis, IN USA

**Keywords:** quality improvement, cerebrovascular disease, transient ischemic attack, quality dashboards, care delivery, audit and feedback, implementation science

## Abstract

**Background:**

Questions persist about how learning healthcare systems should integrate audit and feedback (A&F) into quality improvement (QI) projects to support clinical teams’ use of performance data to improve care quality.

**Objective:**

To identify how a virtual “Hub” dashboard that provided performance data for patients with transient ischemic attack (TIA), a resource library, and a forum for sharing QI plans and tools supported QI activities among newly formed multidisciplinary clinical teams at six Department of Veterans Affairs (VA) medical centers.

**Design:**

An observational, qualitative evaluation of how team members used a web-based Hub.

**Participants:**

External facilitators and multidisciplinary team members at VA facilities engaged in QI to improve the quality of TIA care.

**Approach:**

Qualitative implementation process and summative evaluation of observational Hub data (interviews with Hub users, structured field notes) to identify emergent, contextual themes and patterns of Hub usage.

**Key Results:**

The Hub supported newly formed multidisciplinary teams in implementing QI plans in three main ways: as an information interface for integrated monitoring of TIA performance; as a repository used by local teams and facility champions; and as a tool for team activation. The Hub enabled access to data that were previously inaccessible and unavailable and integrated that data with benchmark and scientific evidence to serve as a common data infrastructure. Led by champions, each implementation team used the Hub differently: local adoption of the staff and patient education materials; benchmarking facility performance against national rates and peer facilities; and positive reinforcement for QI plan development and monitoring. External facilitators used the Hub to help teams leverage data to target areas of improvement and disseminate local adaptations to promote resource sharing across teams.

**Conclusions:**

As a dynamic platform for A&F operating within learning health systems, hubs represent a promising strategy to support local implementation of QI programs by newly formed, multidisciplinary teams.

**Electronic supplementary material:**

The online version of this article (10.1007/s11606-020-06160-y) contains supplementary material, which is available to authorized users.

## INTRODUCTION

To enable data-driven, continuous quality improvement (QI), learning health systems (LHSs) require interfaces that facilitate sharing information throughout fragmented healthcare systems. Readily accessible performance data is critical for LHSs to engage providers and monitor quality of care. Yet to harness the potential for LHSs to be dynamic, adaptive systems, greater attention is needed to understand how clinical teams consume and act on performance data in practice and develop the capacity to learn within local context.^1, 2^ Questions remain about how relevant scientific evidence is presented, acted upon, and spread by those directly involved in implementing QI initiatives and monitoring its impact on quality of delivered care.^3, 4^

The US Department of Veterans Affairs (VA) manages vast amounts of patient-level data collected through electronic health records (EHRs) in a corporate data warehouse (CDW) refreshed on a nightly basis. Although the CDW contains over 2 billion patient encounters, the ability to query and extract relevant data to improve care has lagged behind its capacity for data storage.^5^ Quality and clinical dashboards are technologies which may synthesize multiple sources of data in a concise format.^6^ Dashboards are characterized by data visualization and benchmark targets that support QI.^7^ While research on dashboards in general, and in primary care in particular, has proliferated, little empirical evidence has assessed the pragmatic utility of dashboards and what data is available suggest little impact.^8–10^ Whereas dashboards have shown potential in supporting audit and feedback (A&F), questions persist about how clinical teams use performance data to improve patient care.^11–13^

Approximately 3300 veterans with a TIA are cared for in VA emergency department or inpatient services annually.^14^ TIA care involves multiple services (e.g., primary care, neurology, hospital medicine, emergency medicine, nursing, pharmacy, etc.) across multiple settings (e.g., Emergency Department, inpatient wards, primary care). Given the evidence that TIA patients are at high risk of a recurrent vascular event, such as stroke,^15–17^ and that interventions that deliver timely care can reduce the recurrent vascular event rate by 70%,^18, 19^ there is a clinical imperative to provide timely care to improve outcomes for patients with TIA. Lessons in care coordination learned from a data-driven audit and feedback approach for TIA patients are beneficial to primary care physicians managing other complex conditions, such as congestive heart failure. This evaluation aimed to identify how a virtual “Hub” supported QI among newly formed multidisciplinary teams to improve TIA care quality.

## METHODS

This qualitative evaluation was nested within a prospective, stepped-wedge implementation trial at six diverse VA medical centers across the USA (clinicaltrials.gov: NCT02769338) entitled “Protocol-guided Rapid Evaluation of Veterans Experiencing New Transient Neurological Symptoms (PREVENT).” The trial included a 1-year baseline phase and 1 year of active implementation conducted in three waves (2 facilities per wave). PREVENT was developed based on benchmarking data identifying care quality gaps for TIA patients, interviews with VA staff, and literature regarding effective non-VA TIA programs.^20^ The protocol, described elsewhere,^21^ is comprised of a multifaceted QI program: professional education; quality of care reporting system; QI support and virtual collaborative; clinical programs; and EHR tools and templates. The evaluation was guided by the Consolidated Framework for Implementation Research (CFIR), which can be used to pragmatically conduct formative evaluations in intervention research.^22^ CFIR provides a framework to characterize the context in which the teams utilized the Hub for QI. The use of CFIR provides a common language of domains and constructs derived from implementation theories, models, and evidence. Data was collected as part of the QI trial and analyzed under a study protocol approved by the Institutional Review Board and VA Research and Development Committee.

### Participants and Data Collection

Participants included the team members at the six participating PREVENT facilities and the PREVENT external facilitator. At each facility, participants varied but included staff from primary care (including physicians, nurses, and pharmacists), hospitalist medicine, neurology, emergency medicine, nursing, pharmacy, radiology, vascular surgery, cardiology, ophthalmology, systems redesign, and quality management. The PREVENT site teams were newly formed specifically to engage in acute TIA care QI.

Qualitative data were collected prospectively through structured field notes recorded during kick-off events (conducted at the start of active implementation), at monthly collaborative calls that involved participants at all sites, during in-person interviews at facility visits, and in monthly “office hours” sessions with the external facilitator. The external facilitator was a registered nurse with formal training in facilitation who maintained an activity log that documented all interactions with the PREVENT site team members.

### Intervention: Quality Dashboard

**Figure 1:**
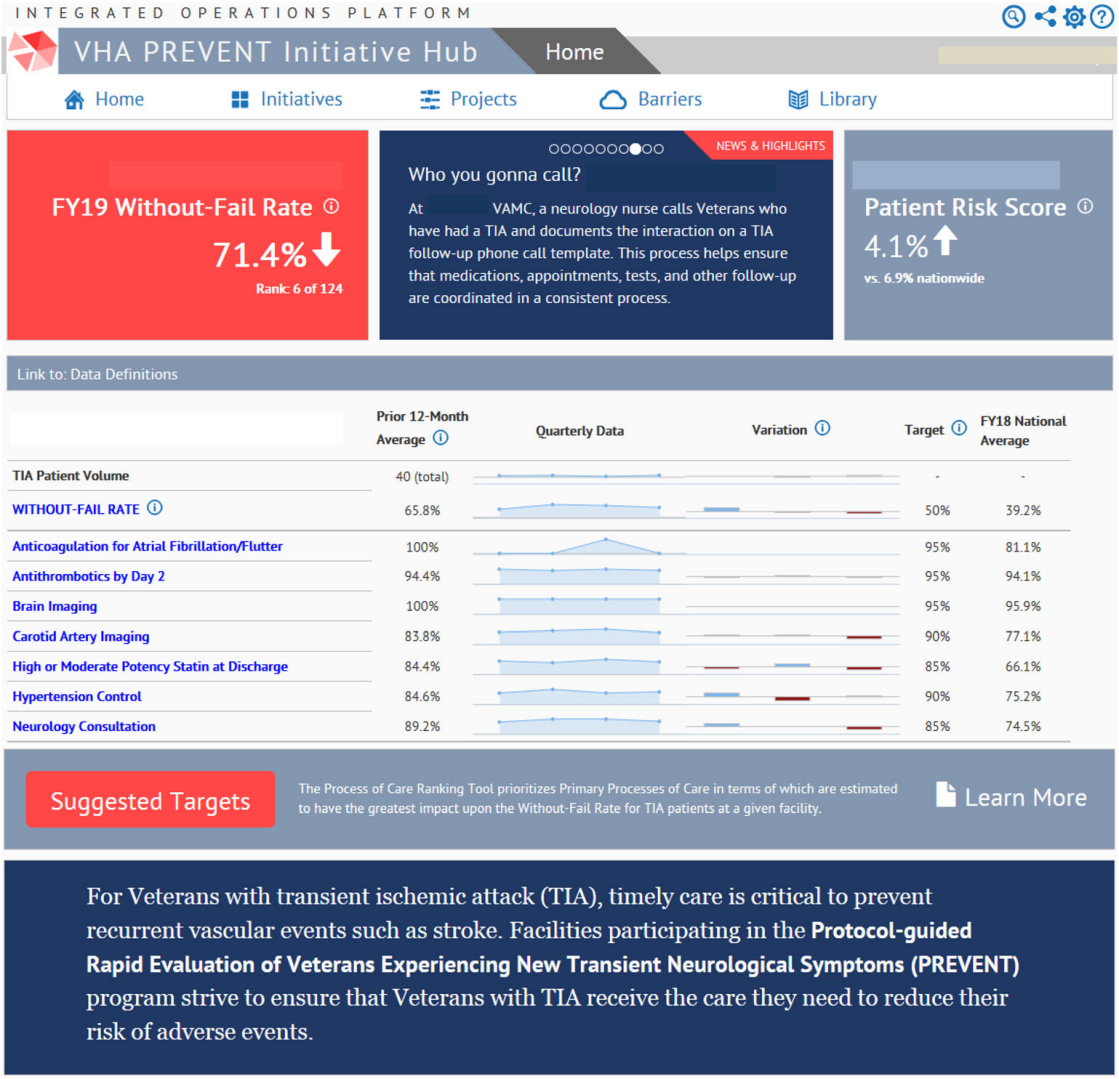
**Screenshot of “quality dashboard” homepage of PREVENT Hub. Note: To maintain anonymity, facility names were removed from this screenshot. In the interface, the Without-Fail Rate in the top-left box (in red) is the current year-to-date WFR and is updated monthly. The WFR rate listed below adjacent to other indicators is the running average over the prior 4 quarters.**

This analysis describes the quality of care reporting system which was developed in partnership with the VA Office of Healthcare Transformation using an existing VA content-management system (“Integrated Operational Platform [IOP]”). We refer to this platform as the “Hub.” The Hub was a web-based platform that resided behind the VA firewall but was distinct from the electronic health record. The web-based Hub had three components:

1. A quality dashboard, tracking facility performance for all patients at that site with a TIA on processes of care,^21^ healthcare utilization, and other aspects of care (see Fig. 1)
2. A library of scientific reports, clinical guidelines, education materials, and EHR tools
3. Interface for monitoring each team’s QI. The Hub served as a virtual “gathering place” and a common frame of reference for participants.

Quality-of-care data were updated monthly and accompanied by suggested targets and national benchmark rates. Beyond predefined metrics, users could also explore how facility performance on metrics of interest varied across time and also for different types of patients through color coding and changes in percentages: patients who presented on weekdays versus weekends, patients admitted versus discharged from the ED to primary care, and patients with or without early neurology consultation. The interface included customizable planning tools for facility QI plans, professional education materials, and a library for sharing tools and resources across facilities.

Hub development began in June 2016. Best practices from the A&F literature were considered during the design phase.^23, 24^ The intervention team collaborated with the VA Office of Healthcare Transformation staff to design the user interface and develop the backend database containing performance data and text-based content. A senior data scientist (LJM) extracted data from the CDW using algorithms to calculate facility-level pass rates on validated performance measures.^25^ The Hub launched July 2017, coinciding with active implementation for the PREVENT site teams, with iterative modifications (Appendix A).

The Hub supported facility-level process improvement/quality monitoring, but it contained tools and resources which could be adopted for patient-level interventions. The Hub was designed as a population-based dashboard for monitoring TIA care quality rather than a real-time decision support tool. Facility champions and team members received a monthly email notification about updated data available on the Hub that included tailored QI suggestions. Moreover, the Hub was an integrated component of the PREVENT QI program. For example, during the kickoff, the facility teams used the Hub to examine their site-specific performance data and to identify processes of care that served as targets for QI activities. Together, the team members developed a site-specific QI plan with short- and long-term QI activities. Those QI plans were uploaded into the Hub with the corresponding performance metrics which allowed for monitoring monthly progress. For example, a facility that was interested in improving hypertension control in the 90-days post-index event might implement a primary care pharmacy-based hypertension project and include metrics related to timely primary care follow-up as well as blood pressure control. Any tools that team members developed during active implementation were added to the Hub library and shared across sites.

The primary outcome of the trial was the “without-fail” rate (WFR), defined as the proportion of patients at the facility who received all of the care for which they were eligible among seven processes of care: anticoagulation for atrial fibrillation, antithrombotics, brain imaging, carotid imaging, high/moderate-potency statins, hypertension control, and neurology consultation. Figure 1 illustrates the Hub dashboard; the upper-left portion of the layout displays the cumulative year-to-date WFR and the facility’s rank among all VA facilities providing TIA care. Changes from the prior month were indicated with arrows. The seven processes included in the WFR were displayed along with 12-month averages, variations in quarterly performance (color coded), and target/national rates. The red “suggested targets” button on the bottom-left of the homepage provided a link to a ranked list of individual processes of care for which there was the greatest gap in care for the greatest number of eligible patients (e.g., only patients with atrial fibrillation were eligible for the anticoagulation process, but the majority of patients were eligible for the hypertension control measure). Tabs along the top of the homepage linked to QI plans and the library of resources.

### Analysis

We evaluated how the teams utilized the Hub to improve their care quality. Hub usage was examined by tracking frequency of visits over time. Qualitative data from interview transcripts and field notes were de-identified, checked for accuracy, and imported into NVivo 12. A team of six analysts carried out an iterative thematic analysis. Open inductive coding generated a codebook. Context-related factors were coded using the applicable constructs from CFIR,^22^ to identify determinants that affect implementation from the participants’ perspective.^26^ Subsequently, individual analysts coded transcripts in teams of two and met to reconcile differences. We report how facilities, teams, and individual staff utilized data from the Hub to improve facility performance.

As a secondary analysis, we evaluated how Hub usage related to completed implementation activities. We ranked participating sites by total Hub visits and dichotomized the sites into “higher” (above the median) and “lower” (below the median) categories. We summed the number of QI activities in each action plan, counted the number of completed QI activities during the 1-year implementation period, and evaluated the proportion of planned activities that were completed. We used a *t* test to assess the two-sided *p* value for the difference in the proportion of activities completed among the higher versus lower Hub usage sites.

## RESULTS

Table 1 describes characteristics of the six facilities, including a description of each facility’s team. A total of 62 interviews were conducted; 74 unique participants were involved in kick-off events, monthly collaborative calls, or office hours. Three overarching themes emerged across seven subthemes: (1) information interface for integrated TIA data, (2) facility context and champions, and (3) team activation as catalyst for “learning to learn.” Themes and subthemes are illustrated with representative quotations.

**Table 1:** Facilities and Data Collected

Site	Facility characteristics			Data		Team description
	VA region	Type	TIA patients (2018)	Interviews (unique)	Unique team participants*	
101	Southeast	Large urban	22	11 (7)	13	Vascular neurologist serving as director of stroke services; multiple engaged pharmacy, telehealth nursing, Emergency Medicine clinicians
102	North Atlantic	Large urban	11	8 (5)	9	Senior neurologist serving as director of stroke services; engaged pharmacy, Chief of Neurology, and Emergency Medicine clinicians
103	Midwest	Large urban	22	9 (6)	11	Vascular neurologist serving as director of stroke services; engaged Emergency Medicine, and internal medicine/hospitalist clinicians; Supportive Chief of Patient Safety
104	Southeast	Medium suburban	37	12 (8)	12	Senior Emergency Medicine nurse clinical champion supported by Internal Medicine leadership
105	Pacific	Medium suburban	23	11 (7)	12	Vascular neurologist serving as director of stroke services; multiple engaged pharmacy, Emergency Medicine, and hospitalist medicine clinicians
106	Southeast	Large urban	47	11 (9)	17	Existing stroke team led by vascular neurologist who was relatively new to the VA; very engaged Emergency Medicine, Pharmacy, nursing education clinicians, supportive Chief of Neurology
Total			162	62 (42)	74	

*During active implementation, each team had core and peripheral members. These figures include attendees on monthly collaborative calls, at kick-off events, and those interviewed

### 1. Information Interface for Integrated TIA Data

The Hub served an information interface and gathering place that enabled clinicians within and across facilities to align practices for TIA care. The Hub addressed three common informatics challenges:^27^ (1a) access to data, (1b) data integration, and (1c) common infrastructure for facility teams and communities of practice.

(1a) *Access to performance data*: Prior to PREVENT, access to facility-level performance data on processes related to TIA care was unavailable. Gaining access to data was critical:

“That lack of data has been extremely difficult to deal with because it’s hard to measure your performance when you don’t have access to the data.” (F103, P1)

“It was super useful. I sincerely wish something like this would’ve existed beyond just like [VA In-Patient Evaluation Center] data for our acute ischemic strokes because I have to manually review those presentations myself for compliance; took me a solid six or eight months to find like an efficient way to map it, graph it, and put it into like a report so that it was like easily understood by our critical care committee/ executive leadership. Having this already done for you, it’s very easy to…. monitor and track compliance.” (F104, P7)

(1b) *Data integration*: The Hub presented facility performance information through a unified data platform. An experienced team, including a data scientist/epidemiologist, physician, and nurse, addressed issues of validation and data quality in response to questions from participating site teams and according to shifts in scientific evidence or local conditions. One facility champion described how data integration and knowing where to target effort are major barriers in QI:

“Being able to see where you are and benchmark yourself against other facilities and then having some like people, you know, dedicated to getting you the information you need about what you need to improve. [They] responded by giving me highly specific data that told us, hey, your problems are neurology consultation … Like a lot of the work is already being done for you, and [data collection] is the hardest part--a challenge that we have in every workgroup that I’m on is people want to bring you their anecdotal stories.” (F103 P6)

(1c) *Common infrastructure for facility teams*: Participants discussed the importance of the Hub as a shared frame of reference for understanding current performance and using timely data to design achievable, specific goals for improving their facility’s WFR and targeting one or more care processes.

### 2. Contextual Factors Affecting Variations in Facility Hub Usage

Across the facilities, Hub usage averaged 18.5 views per month for the first 6 months (111 views) and 25.8 views per month for months 7–12 (155 views). At 5 of the 6 sites, at least half of the implementation team used the Hub regularly. Two Hub usage factors were (2a) participants’ perceptions of design and quality of data on the Hub and (2b) variations in champion activities. Factors that affected Hub usage are described in Table 2, along with key constructs and quotations grouped by relevant CFIR domains, which included intervention design quality, usability, reflecting/evaluating, champions, goals/feedback, and cosmopolitanism.

**Table 2:** Contextual Factors Grouped by CFIR Domains

Domain	Constructs	Representative quotations
Intervention characteristics	• Design quality • Usability	“Love the Hub. I like the fact that we have got all of the listed parameters that we need to follow-- makes everything easy for us to say the one thing we need to focus more attention on is that. The fail rate is great. The library is fantastic.” (F104, P1)
Implementation process	• Reflecting and evaluating • Champions	“It would be helpful as we get these reports back someone says, ‘you know what you got a problem’ (laughing)--the readmission rate—we went from 6.5% [2011] to 11% [2014] and I would be very curious to know what that means and why that, that to me would be an alarming jump.” (F101, P10) “Identifying without fail items gives us a target to shoot at” (F103, P4)
Inner setting	• Goals and feedback	“…the other thing that keeps us motivated is the Hub. The without-fail rate. Just knowing that hey, we are #12 out of 119. Like what can we do to be #1.” (F103, P4) “You could see the impact … I was surprised that just the fact of like admitting them and making sure that we got all of the testing done. … That that did impact patient care. (F101, P5) “Any time that you start to measure something, you kind of galvanize the group … to reach a common goal” (F102, P2)
Outer setting	• Cosmopolitanism	Adaptation of patient education pamphlet between VAMCs: “I used 70% to 80% of [the pamphlet from the Hub] …a lot of the actual material that I used was the same.” (F103, P4)

(2a) *Acceptability of interface and data quality*: Participants expressed satisfaction with the usability and design of the Hub in terms of the quality data dashboard and the resource library. Some participants valued sharing materials via the Hub’s shared library. Most respondents expressed confidence in how data had been compiled; when asked how trustworthy the Hub data was, nearly all participants stated that they trusted the data. However, some participants expressed dissatisfaction with usability and that it was behind a “VA firewall”: “it’s not always straightforward to know what the link—you have to be on a VA computer to be able to do it.” A few individuals expressed skepticism on the reliability of underlying EHR data, attributed lower performance to hospital “coding” problems, or simply indicated they lacked time to visit the dashboard. Some participants accustomed to clinical dashboards expected to be able to drill down to patient data. One user indicated that the Hub lacked a navigation map. Yet one faculty champion (105) explained that “it was helpful to have that [hub] data over time even if it’s not perfectly accurate-- general guidance was helpful,” adding that using a dashboard played a role in sustaining their program.

Participants discussed how they used the Hub based in part on monitoring their facility’s performance. For some providers, their interest in visiting the Hub was strongest following the kick-off, but lessened during active implementation:

“Early on, I was going through it, especially as we were starting to get things organized and starting to pull things together. Those first 2 months, I was going to it fairly routinely… a good couple of times every single week. Since then it’s kind of cooled off.” (102, P3)

Facilities with WFRs that were below target visited the Hub more frequently. As two people separately described, close monitoring motivated teams:

“I look at it at least weekly …I generally always just sort of glance at our without-fail rate—that’s like the encouragement that I need to continue to annoy people … it is a difficult metric to make when you’re digging yourself out of a hole.” (F104, P2)

“You look at our scorecard and it’s gone from all reds to more and more greens. That’s a meaningful progress … (it’s like) that electronic coach--hey, you’re doing great.” (F103, P4)

(2b) *Variations by role and service*: Each of the sites had at least one clinical champion in addition to other site team members. Champions used the Hub differently from other team members: actively monitoring facility performance and communicating trends to team members, reporting to leadership, and updating facility implementation activities on the Hub. Champions varied in terms of when, how, and which data they selected for review. One champion (F101) said: “I look at our statistics—where we are with the … without fail rate.” This champion shared that information verbally with team members during group meetings. At another facility, the champion (F104) paid close attention to monthly updates for each process and also prospectively rounded on the inpatient unit with a checklist intended to ensure that all seven processes were met for each patient—an innovation described as “round crashing.”

All team members drew on tools and resources on the Hub. One champion (F104, P1) explained the educational materials were “very useful for the residents because we’re a big electronic consult place, so this is nice because it’s everything we really need to be updated for in TIA.” Primary care nursing staff used the “TIA Outpatient Processes of Care Checklist” when completing telephone visits after a patient had been discharged from either the Emergency Department or inpatient setting. Pharmacists and nurses tended to use library resources to develop materials for staff and patient education. Pharmacists were primarily interested in materials related to medication management (e.g., hypertension control, statins). In comparison, hospitalists and emergency medicine physicians used sample order sets, EMR templates, and other clinical tools along with scientific evidence supporting TIA processes of care. In specific areas, such as a policy to “admit all TIAs” or to conduct pharmacy education on statin use, subsequent waves benefitted also from lessons learned from wave 1 facilities.

### 3. Team Activation as Catalyst for “Learning to Learn”

This theme included two categories: (3a) using data to activate teams and (3b) developing capacity for team-based learning in the local care system.

(3a) *Activating teams*: Prior to participation in PREVENT, TIA patient care was coordinated informally by individual clinicians. In PREVENT, team members used Hub data to align their QI activities with the larger goal of improving their facility’s overall WFR.

“I use it sort of as a reference because I still go in and do my own review of what is going on; and kind of my own chart reviews to basically confirm information presented on the Hub. … [I] found some discrepancies and a lot of that has to do with coding.” (F105, P3)

Successful facilities identified team meetings as a mechanism for collectively evaluating TIA performance and pinpointing processes for improvement. Some teams established routines that allowed for ongoing reflecting and evaluating. One champion (F102) explained how the Hub facilitated meticulous chart review: “it’s raised the level of consciousness of people … When we [do] fail, you have to look at it on a case-by-case basis and say well, why was it failed?”

(3b) *Capacity for team-based learning*: Participants reflected on how data usage with PREVENT was different than in day-to-day practice, administrative work, or other QI projects. Teams drew on Hub data to make higher-level adaptations for local care delivery. The nature of the data presented on the Hub (e.g., population-level, composite and broken down by process) both elicited and supported investigation of patient cases for points of failure and encouraged process improvement. One person (F103, P2) described how a sense of equanimity and a willingness to further investigate were helpful in effectively using data and “making sense” of the numbers. At this site, the champion worked with the national team to examine individual cases, such as when a patient was not receiving a neurology consultation within 48 h. At one site (F103), performance data from the Hub were shared monthly with facility leadership including temporal graphs and gaps between actual and target performance that were used to plan for adjustments in care delivery. One person (F102, P1) explained that their team was “on the same page” with TIA care: “I definitely get a lot of metrics … But this is kind of a little bit different because there is more perpetuity to the projects—we’re able to come to sustainable action plans.”

### Hub Usage and Quality Improvement Activities Completed

To understand how the Hub supported quality improvement, we examined differences in the number of quality improvement activities completed and found that lower-usage sites completed an average of 7 QI activities which represented 69.0% (standard deviation, 10.2%) of their planned action items and higher-usage sites completed an average of 7.3 QI activities, which represented 75.7% (standard deviation, 6.0%; *p* = 0.383) of their planned items.

## DISCUSSION

The PREVENT Hub, unlike many static dashboards, allowed team members at facilities not only to examine monthly performance data that had been previously inaccessible but also to interact with that data to evaluate change over time, to share resources as facilities engaged in QI, and to foster a sense of inter-facility community. Consistent with the VA’s commitment to the LHS model,^1, 28^ our findings suggest that the Hub actively supported facilities in forming local teams around TIA care with the capacity for learning and adaptive behavior. Site team members utilized the Hub for staff and patient education, benchmarking, and ongoing QI activities; external facilitators used the Hub to help local implementation teams leverage data to target improvement areas in their nascent TIA protocols. Providers cited access to previously unavailable benchmarking data as a key source of motivation to continue improving TIA care. These findings suggest that LHSs can benefit from A&F platforms like the PREVENT Hub when the clinical focus involves complex care delivery; quality data are not readily available; existing resources can be used and re-purposed to help teams provide the highest quality of care (sharing tools, protocols, and other resources prevent teams from having to “reinvent the wheel”); and expertise can be shared across facilities (promoting both learning and professional satisfaction).

Although A&F has been demonstrated to be an effective implementation strategy, multiple reviews report a lack of attention to underlying mechanisms.^23, 29, 30^ In this study, clinical champions used the Hub to reflect actively on current facility performance and then share performance data with others prior to team meetings. Consistent with studies on effective, actionable feedback,^12^ each team devised strategies to address quality gaps, established dedicated time to reflect on performance data, determined whether these trends had ground truth at their facility, and acted to shift current practices at both individual and team levels.^31, 32^ Teams began to engage in “double-loop learning.”^33^ Whereas “single-loop” learning refers to repeated attempts to address problems with existing tools, double-loop learning implies higher-level adaptions that challenge existing behaviors, routines, and underlying goals.^34–36^ Double-loop learning is often paraphrased as “learning to learn” and draws upon the CFIR construct of “Reflecting and Evaluating.” Similar to other CFIR-driven studies, evidence from the present study suggests that setting aside time to evaluate performance in a team setting and to reflect on specific QI goals is a characteristic that distinguishes high- and low-performing facilities.^37^

Facilities in this study faced two challenges that have not received much attention in extant literature. First, facilities had to assemble multidisciplinary teams with individuals unacquainted with one another. Second, although A&F activities often focus on individual or team performance, the WFR measured clinical processes at the facility level and improving the WFR required cooperation between services. Physician and nurse champions explicitly played a boundary-spanning role by developing new social ties with diverse clinicians providing cerebrovascular care.^38, 39, 41^ In this context, the Hub functioned as a “boundary object” (a technological artifact that aids in coordination across multiple social worlds^41^) capable of facilitating cooperation and aligning TIA treatment across an entire facility. Dashboards focused on population or facility-level quality indicators can function as a boundary object that links disparate communities of practice separated by disciplinary understandings, practice, and geography.^42–45^ Importantly, participating facilities largely trusted the data quality and validity due to active facilitation from the national team (i.e., data scientist/epidemiologist, nurse, and physician). Although many clinical dashboards used in primary care provide performance data, few also provide tools and resources that facilitate quality improvement and engagement with colleagues in other services. Sites monitored their performance on specific sub-metrics (i.e., # of patients on nights/weekends, admitted/ED only, etc.) and compared their performance to non-participating peer facilities.

This evaluation has several limitations. First, because the Hub was a core component of a multifaceted QI project, we cannot assess how the Hub itself improved the quality of care apart from qualitative interviews. Second, the small sample restricts generalizability to non-VA settings. For example, some described the inconvenience of accessing the Hub from their non-VA computers, which may be common in other health systems with firewall protection. Third, planned interviews and observations were conducted outside of real-time Hub usage; therefore, we relied on the participants’ recall. However, we included multiple sources of data over time to ensure reliability. Fourth, we held office hours to help site team members troubleshoot problems with the Hub which means that our findings may not be generalizable to projects that lack facilitation. Fifth, participants’ knowledge that their facility performance was being monitored may have influenced their behavior. Finally, given that Hub usage was relatively high, we were not able to evaluate whether hub usage was temporally associated with changes in quality of care. Given that changes in care quality require completion of QI activities that take time to implement (i.e., developing order sets, following by comprehensive training), future studies should be designed to allow temporal assessment of change in quality related to differential dashboard usage.

This evaluation demonstrated how a quality dashboard helped engage local teams, maintain focus on performance targets, and develop a community of practice for a domain of care with little pre-existing clinical or quality infrastructure. The Hub served as a crucial mechanism for supporting the learning healthcare system in practice by enabling implementation teams to learn from their quality improvement data and from each other.

## Electronic supplementary material

ESM 1 (DOCX 24 kb)

## Data Availability

These data must remain on Department of Veterans Affairs servers; investigators interested in working with these data are encouraged to contact the corresponding author.
