# Supplementary material for: Empowering Implementation Teams with a Learning Health System Approach: Leveraging Data to Improve Quality of Care for Transient Ischemic Attack
Source: J Gen Intern Med. 2020 Sep 1;35(Suppl 2):823–31. doi: 10.1007/s11606-020-06160-y (PMC7652965; doi:10.1007/s11606-020-06160-y)
Supplement: Supplementary file 1 — (DOCX 24 kb) [file 11606_2020_6160_MOESM1_ESM.docx]

**Appendix A. Development Timeline for Integrated Operations Platform/”Hub”**

| *Project Phase* | *Key Development Tasks* | *Month* |
| --- | --- | --- |
| Baseline/ Pre-Implementation | Work began toward creating an Excel-based decision support tool and the Integrated Operations Platform/Hub | Sep 2016 |
|  | Iterative and collaborative design process with initial deployment for July kickoffs | Dec 2016 –  July 2017 |
|  | *Without Fail Rates^a^* on home page changed from most recent quarter to FYTD; facility rank added | Aug 2017 |
| Active implementation | Composite score removed from monthly metrics to minimize confusion | Sep 2017 |
|  | *Patient Risk Score^b^* added to home page | Oct 2017 |
|  | Metrics upload automation for project plans | Jan 2018 |
|  | FYTD metrics available through project plans | Feb 2018 |
|  | PREVENT team begins sending email updates regarding TIA performance data | Feb 2018 |
|  | Automation of data updates and layout redesign with 3 patient metrics (*Admitted, Left Against Medical Advice, Patient Risk Score*) | Mar 2018 |
|  | Metrics added: “Visit to Neurology,” “Hypertension Assessment,” “Primary Care visit” (all within 30 days) | Jun 2018 |
|  | Updated color scheme and user improvements following user-centered design evaluation | Jul 2018 |
| ^a^ Without Fail Rate: defined as the proportion of patients with TIA diagnosis that received all of the processes of care for which they were eligible; calculated at facility level based on validated algorithms of EHR data.^45^  ^b^ Patient Risk Score: describes the predicted risk of death within 1 year after TIA, based on both patient characteristics and care processes. | | |

**Appendix B. Glossary of Abbreviations**

**A&F**: Audit and Feedback

**CDW**: Corporate Data Warehouse

**CFIR**: Consolidated Framework for Implementation Research

**EHR**: Electronic Health Record

**IOP**: Integrated Operational Platform

**LHS**: Learning Health System

**PREVENT**: Protocol-guided Rapid Evaluation of Veterans Experiencing New Transient Neurological Symptoms

**TIA**: Transient Ischemic Attack

**QI**: Quality Improvement

**VA**: Veterans Affairs

**WFR**: Without Fail Rate
